# Evidence of Závora Bay as a critical site for reef manta rays, *Mobula alfredi*, in southern Mozambique

**DOI:** 10.1111/jfb.15132

**Published:** 2022-07-23

**Authors:** Michelle Carpenter, Nakia Cullain, Stephanie Kathleen Venables, Yara Tibiriçá, Charles Griffiths, Andrea Denise Marshall

**Affiliations:** ^1^ Department of Biological Sciences University of Cape Town Cape Town South Africa; ^2^ Marine Megafauna Foundation West Palm Beach Florida USA; ^3^ Department of Biology Dalhousie University Halifax Nova Scotia Canada; ^4^ Departamento de Biología, Facultad de Ciencias del Mar y Ambientales Universidad de Cádiz Cádiz Spain

**Keywords:** mark recapture, Mobulidae, photo‐ID, population demography, robust design, southern Africa

## Abstract

The largest known reef manta ray (*Mobula alfredi*) population in Africa has been monitored for more than 20 years at several locations on the coast of the Inhambane Province in southern Mozambique. Nonetheless, before this study, little had been reported on the population dynamics of *M. alfredi* from Závora, a remote bay in the region. Photographic mark‐recapture was used to investigate the size and structure of *M. alfredi* that aggregate at “Red Sands,” a reef cleaning station in Závora Bay. An 11 year photographic data set was used to identify 583 *M. alfredi* individuals between 2010 and 2021. More than half of *M. alfredi* individuals were resighted at least once, with most encounters (up to 18 for one individual) occurring during the peak sighting period in July–November each year. An even sex ratio was observed, 44% females and 50% males, with no significant difference in resightings between the sexes. Pollock's robust design population models were used to estimate annual abundance, emigration, annual apparent survival and capture probability at Red Sands from July to November over a 6 year period (2016–2021). Abundance estimates varied year to year, ranging from 35 (95% c.i. [30, 45]) up to 233 (95% c.i. [224, 249]) *M. alfredi* individuals. Given the seasonal affinity of *M. alfredi* observed at Red Sands, this study highlights the importance of understanding fine‐scale site use within the larger home range of this population to develop local management strategies.

**SIGNIFICANCE STATEMENT**

The purpose of this study is to better understand areas of critical habitat for reef manta rays in southern Mozambique, specifically, the use of a cleaning station called “Red Sands” (RS) in Závora Bay in the Inhambane Province. This is important because cleaning stations, areas on reef where fish remove parasites or dead skin off of a “client” animal, are sites that manta rays have been found to repeatedly return to. The authors found a large seasonal abundance of manta rays at RS, displaying the importance of this site for the greater southern Mozambique population. As RS is currently unprotected, the study demonstrates the need for immediate site‐specific protection.

## 1 INTRODUCTION

Understanding population trends of large, migratory, marine species can be challenging because of the difficulty in assessing habitat use within their estimated home range. Knowledge of fine‐scale visitation patterns at specific locations through focused site‐specific studies can be beneficial to understanding population dynamics, identifying priority areas for protection and developing local management regimes. The use of photographic mark‐recapture is rapidly co‐evolving in parallel with both technological advancements *in camera* equipment, open‐source websites, algorithm development and growth in citizen science initiatives (*e.g*., public data submissions and increased internet access), making it an increasingly powerful tool for long‐term population monitoring of wide‐ranging species across many taxa and habitat types (Karanth, 1995; Dala‐Corte *et al*., 2016; Marshall & Pierce, 2012; McConkey, 1999; Schofield *et al*., 2008; Towner *et al*., 2013; Wiirsig & Jefferson, 1990). Predictable aggregations at certain sites allow snapshots of population sizes, trends and movement patterns of these elusive species.

Manta rays (order Myliobatiformes) are wide‐ranging, pelagic filter feeders that aggregate at inshore reefs, islands or seamounts (Couturier *et al*., 2012; Murie *et al*., 2020; Harris *et al*., 2020). Their aggregations can be directly related to foraging, or at cleaning stations in close proximity to feeding grounds, to solicit cleaning services by symbiotic fish and sometimes engage in social or reproductive interactions (Couturier *et al*., 2011; Limbaugh, 1961; Stevens, 2016). The unique and stable ventral markings of individual rays have facilitated photo‐identification (photo‐ID) studies at these aggregation sites, providing the foundation for manta ray research in many locations across the globe (*e.g*., Couturier *et al*., 2014; Deakos *et al*., 2011; Germanov *et al*., 2019; Harris *et al*., 2020; Homma *et al*., 1999; Kumli & Rubin, 2008; Marshall *et al*., 2011; Stevens, 2016). This technique has been used to assess home range (Deakos *et al*., 2011; Kashiwagi *et al*., 2011), longevity (Clark, 2010; Couturier *et al*., 2014; Kashiwagi, 2014; Rubin, 2002), migration patterns (Armstrong *et al*., 2019; Germanov & Marshall, 2014), site affinity (Couturier *et al*., 2011; Germanov *et al*., 2019; Marshall *et al*., 2011), reproductive ecology (Deakos *et al*., 2011; Marshall & Bennett, 2010a; Stevens, 2016) and estimating abundance (Beale *et al*., 2019; Couturier *et al*., 2014; Venables, 2020). Regional reef manta ray [*Mobula alfredi* (Kreft, 1868)] photo‐ID databases vary substantially in the total number of individuals identified over time, ranging from populations in the low hundreds (Axworthy *et al*., 2019; Carpentier *et al*., 2019; Deakos *et al*., 2011; Kashiwagi, 2014; Peel, 2019) to those in the thousands (Armstrong *et al*., 2019; Stevens, 2016; Venables, 2020).

Mark‐recapture population modelling provides a tool through which photo‐ID data can be analysed to provide abundance estimates. Predictable patterns in the use of critical habitats, such as cleaning stations and feeding locations, make *M. alfredi* a suitable candidate for this technique (Couturier *et al*., 2011; Venables, 2020). With an initial photo of the ventral spot patterning signifying an individual's “mark” and subsequent photos representing their “recaptures,” these data are further analysed through models to estimate population parameters (Couturier *et al*., 2014; Grusd *et al*., 2019). Previous mark‐recapture studies of *M. alfredi* have used various model types [*i.e*., Cormack–Jolly–Seber (CJS); Petersen's method; Pollock's robust design (PRD)] (Couturier *et al*., 2014; Deakos *et al*., 2011; Kitchen‐Wheeler *et al*., 2012; Marshall *et al*., 2011). More recently, the robust design has proven useful, with the ability to account for temporary emigration and capture heterogeneity, which are inherent in mobile marine species (Couturier *et al*., 2014; Venables, 2020). PRD models are characterised by marginal dependence between abundance and survival estimators, as well as estimation of temporary emigration, all of which improve the precision of population estimates and interpretations of the relationship between abundance and survival (Grusd *et al*., 2019; Kendall *et al*., 1995; Pollock, 1981; Pollock *et al*., 1990).

A 16 year study in Mozambique documented the largest photo‐identified population of *M. alfredi* in Africa, with the number of identified individuals currently reported to be 1209 (Marshall *et al*., 2011; Venables, 2020). With increased annual sampling effort, *M. alfredi* continues to exhibit long‐term affinity to monitored cleaning stations in Mozambique; some individuals returning to the same sites for more than 15 years (Venables, 2020). Estimations of annual abundances in the Praia do Tofo region peaked at 836 individuals in 2004–2005 (Venables, 2020). However, sightings declined of up to 88% between 2003 and 2011 (Rohner *et al*., 2013), and estimates of only 100 individuals sighted in Tofo after 2013 (Venables, 2020) have raised immediate concern about the health of this population. *M. alfredi* is listed on the IUCN Red List as Vulnerable (Marshall *et al*., 2018) and is globally threatened from direct harvesting of the gill plates for the Asian market, by‐catch, destructive fisheries methods and coastal development, which in turn leads to increased boat strikes, habitat loss and pollution (Couturier *et al*., 2012; Croll *et al*., 2016; Fernando & Stewart 2021; Lawson *et al*., 2017; O'Malley *et al*., 2016). Monitoring of populations of this threatened species is thus crucial for future IUCN Red List assessments and further development of local management actions, such as the designation of new marine‐protected areas and regulations surrounding fisheries and tourism operations.

Although the Tofo region has been consistently monitored since 2003, an aggregation site for *M. alfredi* 90 km south in Závora has not yet been assessed. The authors of this study aim to better understand this specific aggregation and assess its importance for the larger southern Mozambican population of *M. alfredi*. They use an 11 year photo‐ID database of individuals to describe population demographics, site affinity and resightings data. Using PRD mark‐recapture modelling, they estimate annual abundance and population parameters including apparent survival, emigration and recapture probability at RS between 2016 and 2021. The findings can be used to inform the development of local conservation strategies and guide the design and implementation of spatial management approaches, such as marine‐protected areas, in the Závora Bay region of the Inhambane coastline.

## 2 MATERIALS AND METHODS

### 2.1 Study site

**FIGURE 1:**
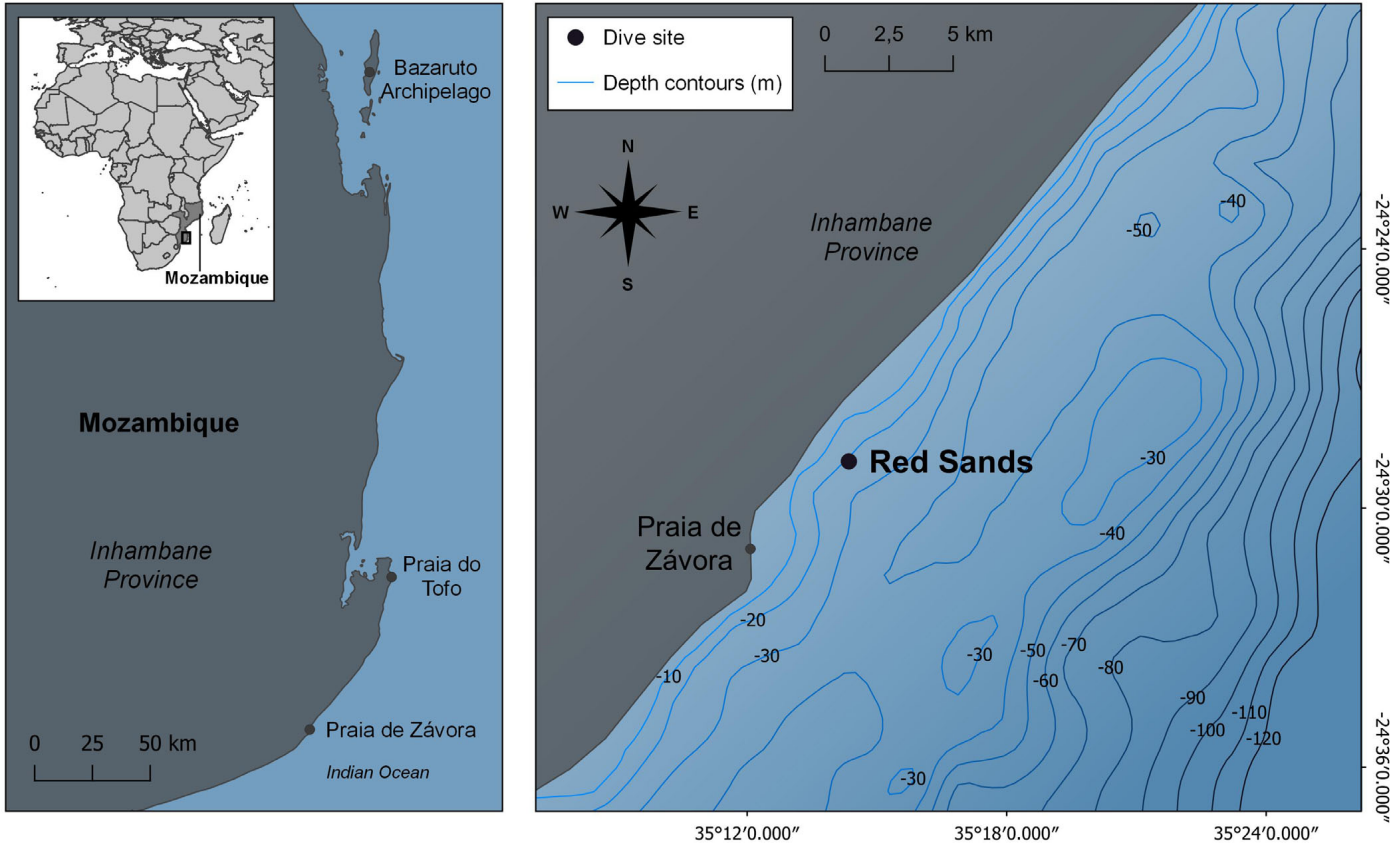
Study map of Závora Bay showing the location of the Red Sands cleaning station and the bathymetry of the bay

In Mozambique, *M. alfredi* is most commonly encountered in the coastal waters of the Inhambane Province, particularly from the Bazaruto Archipelago in the north to Závora in the south (Figure 1). This 350 km stretch of coastline joins a narrow continental shelf that experiences regular upwelling events, resulting in productive waters that attract several planktivorous species, including whale sharks, *Rhincodon typus*, giant manta rays, *Mobula birostris*, and shortfin devil rays, *Mobula kuhlii* (Rohner *et al*., 2013; Quartly & Srokosz, 2004). *M. alfredi* has been monitored in Závora since 2010; nonetheless, because of limited resources, the remoteness of this location and minimal tourism/recreational diving, a comprehensive sampling design was not initiated until 2016. Red Sands (RS) is a rocky reef with scattered corals and sponges, at 12–18 m depth, *c*. 3 km offshore. The site is characterised by variability in environmental conditions: with horizontal visibility ranging 1–20 m, various levels of current and surge, and sea temperatures ranging from 16 to 23°C in the winter and up to 27°C in the summer (Cullain, unpubl. data).

### 2.2 Sampling effort and design

Photographic sampling by trained researchers was conducted at RS on SCUBA diving between 2010 and 2021. Weather, logistical limitations and COVID‐19 restrictions prohibited consistent, daily sampling effort throughout and between years. During each survey, teams of two to eight divers swam a transect that covered all monitored cleaning stations that make up RS. Upon encountering an individual *M. alfredi*, a photo of the unique markings on the ventral surface was taken. Sex was determined by the presence of external claspers for males and absence for females (Marshall & Bennett, 2010a). The authors assessed male maturity by the size of reproductive organs, individuals being considered adult once the claspers extended past the posterior edge of the pectoral fins (Marshall & Bennett, 2010a). Female maturity was determined by the observation of pregnancy (when the abdomen was clearly expanded), or the presence of reproductive scars usually on the left pectoral fin (99% lateralisation: Marshall & Bennett, 2010a). A female that was not noticeably pregnant, nor had mating scars, was recorded as unknown maturity. The total number of identified and unidentified individuals were pooled for each day of sampling. Resightings of an individual were recorded when identified more than 24 h after the last sighting.

The authors used photo‐ID data collected from 2010 to 2021 to assess population demographics. Mean counts of individuals or time periods between sightings were calculated to assess the number and resightings of males and females (± standard deviation). Mark‐recapture models require consistent survey effort and because of the nature of the current data set, only the most recent 6 years fit these criteria (82% of the total identifications). Therefore, data collected at RS during the 5 month peak season (July–November) of 2016–2021 were included in the PRD modelling, resulting in six primary periods (years) and 29 secondary periods (months; Table 1). Of the total 583 *M. alfredi* individuals catalogued for Závora, 401 were photo‐identified at RS between 2016 and 2021 and were included in the PRD. Of these, individuals of undetermined sex (*n* = 18) were removed for the final PRD analysis that included sex as a covariate.

**TABLE 1:** Sampling effort (minutes) during primary periods (years) and secondary periods (months) used for Pollock's robust design of *Mobula alfredi* at Red Sands, Závora, Mozambique

Secondary period	2016	2017	2018	2019	2020	2021
July	68	459	584	833	182	420
August	318	445	570	1252	569	790
September	194	771	725	615	609	828
October	95	481	632	469	44	471
November	0; omitted for PRD	696	456	369	72	333

### 2.3 Mark‐recapture analysis

The authors used a PRD with Huggins’ estimator to analyse 6 year photographic mark‐recapture data of *M. alfredi* at RS, Závora (Huggins, 1989; Pollock *et al*., 1990). Models were assembled using package “RMark” (Laake, 2013) in R Version 4.1.2 (R Core Team, 2021), the R interface to programme MARK (Cooch & White, 2006; White & Burnham, 1999). The six peak seasons were selected as primary periods because of higher *M. alfredi* sightings; each winter season had five monthly secondary periods (July–November), except 2016 which had four (July–October), because of no survey effort in November 2016. *Mobula alfredi* sightings were lower in December–June; therefore, these months were excluded to allow adequate time between primary periods to detect fluctuations in the population (Kendall, 1999; Silva *et al*., 2009). Few *M. alfredi* individuals (*n* = 21) were sighted at other reefs in Závora, but never encountered at RS; therefore, these individuals were excluded from the present study (Supporting Information Table S1).

PRD models have the following assumptions: all ventral markings on *M. alfredi* individuals were unique and remained stable over time, the population was open to immigration, emigration, natality and mortality between years, full closure within the aggregation months and equal survival probability on all individuals (Cooch & White, 2006; Kendall *et al*., 1995; Smith *et al*., 2013; Williams *et al*., 2002). Closure was not assumed at RS specifically; rather the authors of this study assumed that the individuals encountered at RS remained in the Závora Bay region during these time periods and were thus recaptured at RS.

The authors evaluated apparent survival between primary periods as time‐constant *φ*(·), time varying *φ*(*t*) and with a group effect for sex *φ*(sex). Models including time‐varying survival consistently yielded inestimable parameters. The authors deemed it appropriate to exclude time‐varying survival from the final model set due to the longevity of *M. alfredi* once mature; previous studies on *M. alfredi* populations found survival estimates close to 1.0 between years (Couturier *et al*., 2014; Kitchen‐Wheeler *et al*., 2012). The temporary emigration parameter represents the probability of present individuals in the population being absent for capture in a specific period (Kendall *et al*., 1997). This was assessed as Markovian (*γ*′ and *γ*″), random *γ* (*γ*′ = *γ*″) or none (*γ*′, *γ*″ = 0). Capture *p* and recapture *c* probabilities were modelled as time‐constant *p*(·), time‐varying per year *p*(*y*) and with effects of sampling effort *p*(*s*). Equal capture and recapture probability (*p* = *c*) was excluded from the final candidate model set due to inestimable parameters resulting from the variability of encounters per secondary period. Parameter estimates were model averaged based on the model weight. The authors evaluated the confidence interval (c.i.) and standard error (s.e.) of each estimated parameter. The PRD analysis was subsequently conducted on the same data with pooled sexes to yield numbers for total population abundance across the primary periods. AICc was used to evaluate the best model that fitted the data, determined by the smallest AICc value (Burnham & Anderson, 2004). A Mann–Whitney *U*‐test was conducted using the “exactRankTests” R package to analyse the effect of sex on the total number of recaptures during the study period, with individuals of undetermined sex excluded from the analysis (Hothorn & Hornik 2021). Significance was accepted at *P* < 0.05.

### 2.4 Lagged identification rates

The authors used lagged identification rates (LIR), the probability of resighting an individual after a given time lag, to estimate site use of *M. alfredi* at RS (Whitehead, 2001). The SOCPROG 2.9 programme (Whitehead, 2009), specifically the “movement analysis” module, was used. The authors compared observed individual sighting data from 2016 to 2021, when there was consistent survey effort, to several exponential mathematical models that represented various habitat use scenarios, including permanent residency, emigration and mortality, emigration and reimmigration, emigration and reimmigration with mortality and a cyclical pattern of appearance. The quasi‐AIC values were used to select the best supported model due to the overdispersion of the data (Whitehead, 2007). Data were bootstrapped 100 times, with 1000 maximum evaluations, to estimate the standard error and parameter precision (Buckland & Garthwaite, 1991; Whitehead, 2001).

## 3 RESULTS

### 3.1 Population demographics

**FIGURE 2:**
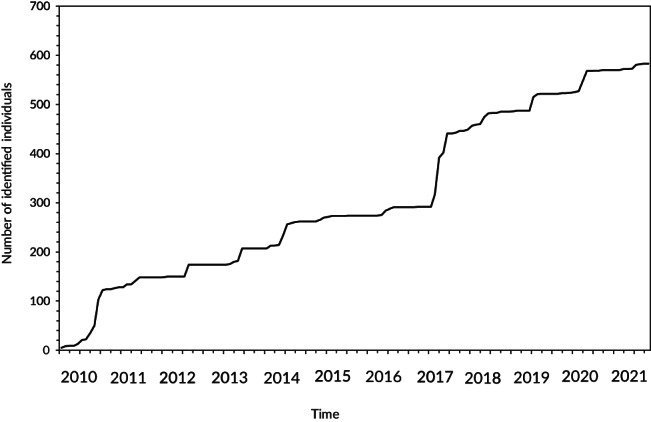
Discovery curve of identified *Mobula alfredi* individuals from 2010 to 2021 in Závora, Mozambique

**FIGURE 3:**
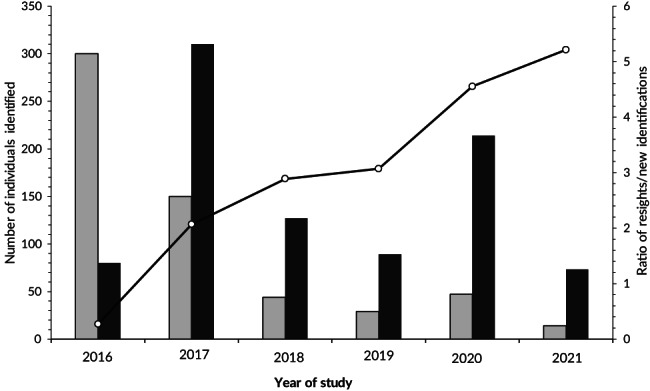
Total number of newly identified *Mobula alfredi* (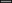) and the total number of resights (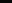) in each primary period of the study, and the ratio of resights/new at Red Sands (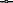), in Závora, Mozambique

Sampling effort at RS ranged from 0 to 37 dives per month, with one or two dives of 44–72 min duration conducted per day, resulting in a monthly sampling effort of between 44 and 1252 min (Table 1). The number of *M. alfredi* individuals in the photo‐ID database increased throughout the study period with large numbers of new identifications between 2010–2011 and 2017–2018 (Figure 2). Until 2016, the number of newly identified *M. alfredi* surpassed resights, and after 2017, the number of resighted individuals exceeded new IDs (Figure 3). An average of three individuals (±4.29) and up to 61 individuals (10% of the photographed population) in a single day were identified visiting RS during peak season (2016–2021; *n* = 274 total identifications in one July–November season).

**FIGURE 4:**
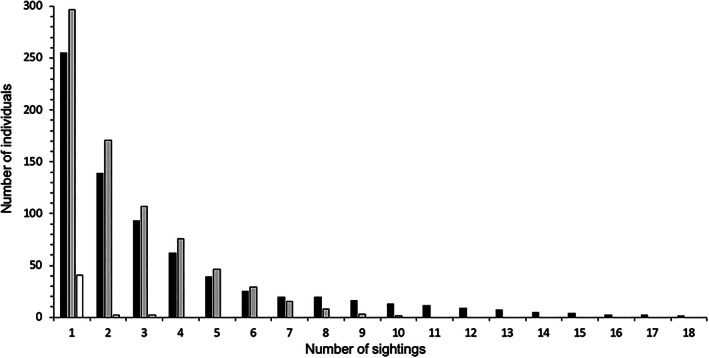
Total number of *Mobula alfredi* identified at Red Sands in Závora, Mozambique: female (black 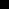), male (grey 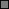) and undetermined (white 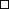)

Between 2010 and 2021, the authors recorded 1509 encounters of 583 individual *M. alfredi* in Závora Bay. More than half, 54% (*n* = 312), of these were resighted at least once; 57% (*n* = 331) of individuals were seen only within a single year and 43% (*n* = 252) across multiple years. The mean time interval between initial and subsequent sightings was 455 days (±694), with 10 individuals recorded with a resighting interval of 1000 days or more, and a maximum of 10.9 years (3996 days) between resightings. The population exhibited an even sex ratio, whereby 44% (*n* = 255) were females, 50% were males (*n* = 295) and sex could not be determined for 6% (*n* = 33). There was no significant difference in the mean number of sightings between females and males 2.82 (±2.34) and 2.53 (±2.34), respectively (Mann–Whitney *U*‐test; *P* = 0.7981). Although more males than females were resighted (males, *n* = 171; females, *n* = 139) in Závora, individuals in the database that were sighted six or fewer times consisted of mostly males, whereas individuals sighted seven times or more during the study period were almost all females (Figure 4). Only mature females had more than 10 sightings during the study period, with the most resighted individual identified 18 times.

The authors recorded 44 pregnancies across 36 females during the 11 year study period. Five individuals were observed to be pregnant on more than one occasion, with a mean postpartum interval of 33.4 (±8.8 months; Supporting Information Figure S1). About 56% (*n* = 326) of *M. alfredi* were defined as mature, 49% (*n* = 288) being males and 7% females (*n* = 38), although for most females maturity could not be determined (*n* = 250).

### 3.2 Robust design

**FIGURE 5:**
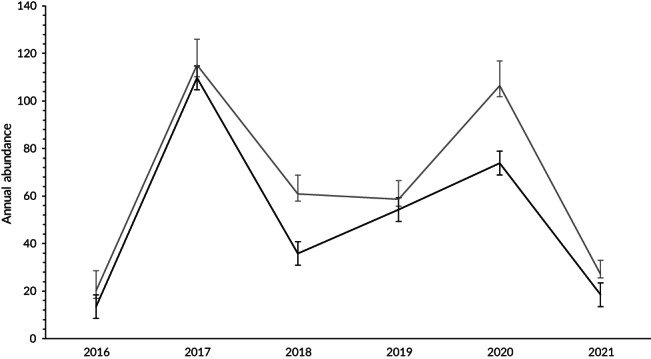
Estimates of yearly abundance of *Mobula alfredi* (*N*) ± 95% c.i. at Red Sands in Závora, Mozambique from 2016 to 2021, estimated from the best‐fit model (φ𝑆𝑒*x* 𝛾″𝑀 𝛾′𝑀 𝜌𝑠 =𝑐()) with sex as a covariate (a; grey = male, black = female)

Eighteen candidate models were evaluated in the PRD analysis. Models that integrated Markovian emigration, with capture probability varying by sampling effort, were best supported (Table 2). The best supported PRD model consisted of sex‐dependent survival, Markovian temporary emigration and an effect of sampling effort on capture probability (Table 2). Annual apparent survival was estimated higher for males than females, at 0.848 (0.09; 95% c.i. [0.597, 0.954]) and 0.823 (0.08; 95% c.i. [0.602, 0.935]), respectively (Supporting Information Table S2). Capture probability dependent on sampling effort fluctuated between primary periods, with the highest in 2020 (0.69; 95% c.i. [0.60, 0.76]) and lowest in 2016 (0.16; 95% c.i. [0.14, 0.18]) (Supporting Information Table S2). Overall annual abundances ranged from 35 (95% c.i. [30, 45]) in 2016 to 233 (95% c.i. [224, 249]) in 2017 (Table 3). Differences in annual abundance estimates were marginal for males and females, at 20–115 and 13–110, respectively (Figure 5).

**TABLE 2:** Selection of Pollock's robust design (*n* = 18) candidate models for estimations of population size (*N*), survival (*φ*; constant or sex varying), temporary emigration (*γ″* and *γ′*; Markovian, random or none), capture (*p*) and recapture (*c*) probabilities (constant, with response to capture, varying by year or varying by sampling effort) of *Mobula alfredi* individuals that use Red Sands in Závora, Mozambique

Model	Rank	npar	AICc	ΔAICc	Model weight	Deviance
φ_*Sex*_ *γ″*_*M*_ *γ′*_*M*_ *ρ*_*s*_ = *c*()	1	15	4137.92	0.00	0.846	5047.43
φ. *γ″*_*M*_ *γ′*_*M*_ *ρ*_*s*_ = *c*()	2	13	4141.32	3.40	0.154	5039.88
φ_*Sex*_ *γ″*_*R*_ = *γ′*_*R*_ *ρ*_*s*_ = *c*()	3	7	4195.45	57.53	0.000	5113.87
φ. *γ″*_*R*_ = *γ′*_*R*_ *ρ*_*s*_ = *c*()	4	5	4198.89	60.97	0.000	5121.37
φ_*Sex*_ *γ″*_0_ = *γ′*_0_ *ρ*_*y*_ = *c*()	5	9	4236.52	98.60	0.000	5150.85
φ_*Sex*_ *γ″*_*R*_ = *γ′*_*R*_ *ρ*_*y*_ = *c*()	6	10	4238.52	100.65	0.000	5150.85
φ. *γ″*_0_ = *γ′*_0_ *ρ*_*y*_ = *c*()	7	7	4240.40	102.49	0.000	5158.82
φ. *γ″*_*R*_ = *γ′*_*R*_ *ρ*_*y*_ = *c*()	8	8	4242.44	104.53	0.000	5158.82
φ_*Sex*_ *γ″*_*M*_ = *γ′*_*M*_ *ρ*_*y*_ = *c*()	9	16	4242.52	104.60	0.000	5142.40
φ. *γ″*_*M*_ *γ′*_*M*_ *ρ*_*y*_ = *c*()	10	14	4245.91	107.99	0.000	5149.95
φ_*Sex*_ *γ″*_0_ = *γ′*_0_ *ρ*_*s*_ = *c*()	11	6	4295.44	157.52	0.000	5215.90
φ. *γ″*_0_ = *γ′*_0_ *ρ*_*s*_ = *c*()	12	4	4299.05	161.13	0.000	5223.56
φ_*Sex*_ *γ″*_*R*_ = *γ′*_*R*_ *ρ* ⋅ = *c*()	13	5	4352.64	214.73	0.000	5275.13
φ. *γ″*_*R*_ = *γ′*_*R*_ *ρ* ⋅ = *c*()	14	3	4356.10	218.18	0.000	5282.63
φ_*Sex*_ *γ″*_*M*_ *γ′*_*M*_ *ρ* ⋅ = *c*()	15	11	4359.07	221.16	0.000	5269.31
φ. *γ″*_*M*_ = *γ′*_*M*_ *ρ* ⋅ = *c*()	16	9	4362.09	224.17	0.000	5276.43
φ. *γ″*_0_ = *γ′*_0_ *ρ* ⋅ = *c*()	17	4	4364.08	226.16	0.000	5288.59
φ. *γ″*_0_ = *γ′*_0_ *ρ*_*s*_ = *c*()	18	2	4367.73	229.81	0.000	5296.27

**TABLE 3:** Population size (*N*) for males, females and overall *Mobula alfredi* at Red Sands in Závora, Mozambique, from the weighted average of the best‐fit models, and the number of uniquely photo‐identified individuals between July 2016 and November 2021

Sex	Method	Year	Weighted average	s.e.	95% c.i.
Male	PRD	2016	20	2.71	17–28
		2017	115	3.79	110–126
		2018	61	2.55	58–69
		2019	59	2.50	56–66
		2020	106	3.60	102–117
		2021	27	1.61	26–33
	Photo‐ID	2016–2021	215		
Female	PRD	2016	13	2.19	11–21
		2017	110	3.67	105–120
		2018	36	1.88	34–42
		2019	54	2.38	52–62
		2020	74	2.87	70–83
		2021	18	1.31	17–24
	Photo‐ID	2016–2021	168		
Overall	PRD	2016	35	3.63	30–45
		2017	233	6.16	224–249
		2018	102	3.51	98–112
		2019	114	3.77	109–125
		2020	185	5.22	178–199
		2021	49	2.24	46–56
	Photo‐ID	2016–2021	401		

### 3.3 Lagged identification rates

**FIGURE 6:**
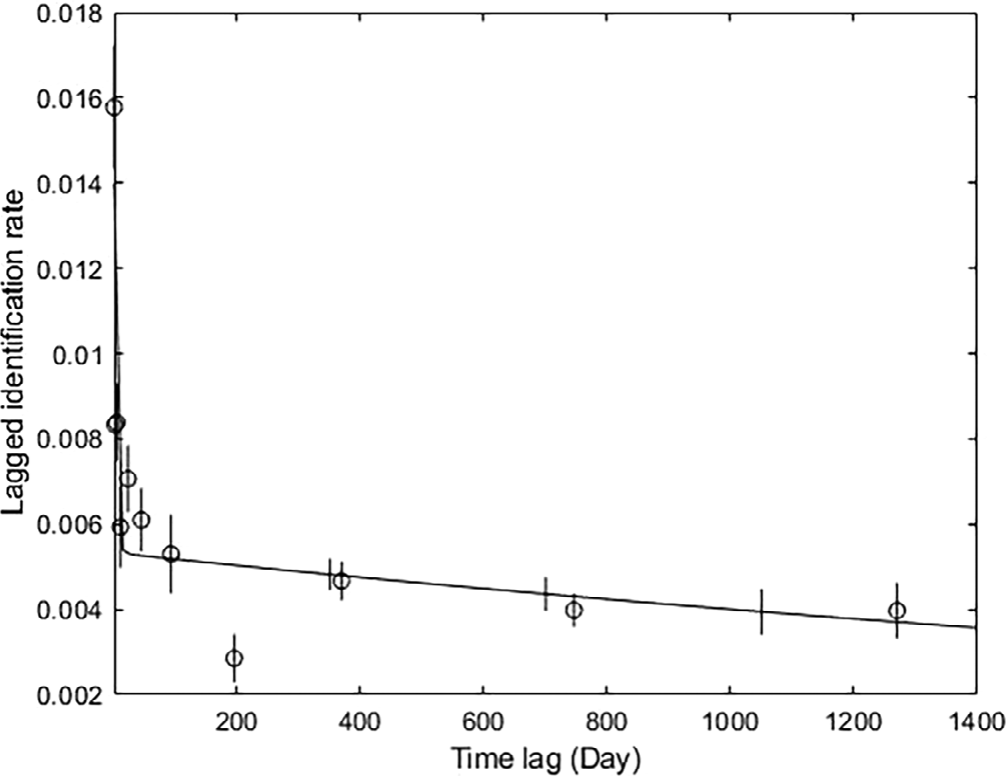
Empirical data (mean ± s.e.) for the lagged identification rate, the probability of re‐identifying *Mobula alfredi* in Závora Bay, Mozambique, over increasing time periods, with fitted emigration plus reimmigration plus mortality model

The best fit LIR models were F and H, which were practically equivalent at <2 based on the ΔQAIC values (Supporting Information Table S3). Nonetheless, model H made biological sense for the data, which incorporated immigration, reimmigration and permanent emigration and/or mortality (Table 4). Approximately 58 individuals (s.e. = 16.19, 95% c.i. [35.37, 95.71]) were estimated to be present in the study area on a given day. *Mobula alfredi* individuals had a mean residence time of 4 days (s.e. = 27.82, 95% c.i. [1.53, 80.02] days), with 10 days (s.e. = 415.81, 95% c.i. [5.22, 155.49] days) away from the study area. Permanent emigration and/or mortality was estimated at 0.00029 (s.e. = 0.00029, 95% c.i. [−0.00024, 0.00070]). The plotted LIR curve decreased rapidly from the date after identification indicating that most individuals were transient to RS, with temporal annual use of RS (Figure 6). The plotted LIR curve then levels and decreases until the end of the study period suggesting emigration and subsequent return and/or return to the area each season. The declining rate of the LIR displays individual dispersal and the shape of the curve indicates a short residency period at the aggregation site, with reimmigration at a later stage by a proportion of the individuals (Whitehead, 2009).

**TABLE 4:** Model selection for lagged identification rate of reef manta rays in Závora Bay, Mozambique (2016–2021)

Model	Model description	ΔQAIC
A	Closed (1/*a*1 = *N*)	89.60
B	Closed (*a*1 = *N*)	89.60
C	Emigration/mortality (*a*1 = emigration rate; 1/*a*2 = *N*)	46.05
D	Emigration/mortality (*a*1 = *N*; *a*2 = mean residence time)	46.05
E	Emigration + reimmigration (*a*1 = emigration rate; *a*2/(*a*2 + *a*3) = proportion of population in study area at any time)	28.15
F	Emigration + reimmigration (*a*1 = *N*; *a*2 = res time in; *a*3 = res time out	0.95
G	Emigration + reimmigration + mortality	9.93
H	Emigration + reimmigration + mortality *a*1 = *N*; *a*2 = res time in; *a*3 = res time out; *a*4 = mortality	0.00
I	Cyclical *a*1 × cos (*a*2 × *td*) + *a*3	93.56

## 4 DISCUSSION

In this study, the authors describe a seasonal, site‐specific aggregation of *M. alfredi* at RS, an aggregation site in Závora Bay, showing this site to be an important regional habitat for the species. Half of the 583 identified individuals displayed affinity to this location (54% resighting rate) despite belonging to a larger, wide‐ranging population (Venables *et al*., 2021). A total of 312 individuals returned to RS on multiple occasions, up to a maximum of 18 times during the 11 year study period, with no difference in site use between the sexes. Estimated annual abundance at RS ranged from 35 to 233 individuals during the July–November season. This seasonal peak in abundance combined with the resighting rate of individuals reflects the seasonal importance of the Závora region for a proportion of the larger *M. alfredi* population in southern Mozambique. Compared with other monitored *M. alfredi* populations that found constant survival to be ≥0.9 (Deakos *et al*., 2011; Couturier *et al*., 2014), there was a lower apparent survival (males, 0.848; females, 0.823) at RS, suggesting transience during periods when conditions are not favourable for visitation to Závora Bay. This was further supported by an average residence time of 4 days estimated using LIR.

*Mobula alfredi* habitat use varies spatially and temporally at other well‐studied locations (Armstrong *et al*., 2020; Dewar *et al*., 2008), which is also evident in the findings. Site affinity has been globally reported for *M. alfredi*, with individuals consistently returning to cleaning station reefs over long periods of time, up to 30 years (Couturier *et al*., 2014; Couturier *et al*., 2018; Dewar *et al*., 2008; Venables *et al*., 2020). The results show that *M. alfredi* presently aggregate at one shallow reef in Závora, rather than a collection of deeper reefs (25–30 m) as documented in Tofo (Marshall *et al*., 2011; Venables, 2020). Higher resighting rates were found at RS (54%) compared with cleaning stations in eastern Australia (Couturier *et al*., 2011) and Tofo (Venables, 2020), but less than Indonesia, Hawaii and the Maldives (Couturier *et al*., 2011; Deakos *et al*., 2011; Germanov *et al*., 2019; Harris & Stevens, 2021). The authors found seasonal peaks in sightings at Závora, as opposed to year‐round sightings at other identified *M. alfredi* aggregations in the Inhambane Province (Venables, 2020). Island populations of *M. alfredi* in the western Indian Ocean also exhibit year‐round site use, with seasonally driven peaks related to monsoon winds (Peel *et al*., 2019; Stevens, 2016). Although oceanic processes such as monsoonal shifts, seasonal‐driven currents and tides affect *M. alfredi* site use in the Komodo National Park, Indonesia and eastern Australia, ontogenetic patterns were found to influence habitat use in Nusa Penida, Indonesia, the Gulf of Mexico and Hawaii, and the authors suggest these could be potential drivers of *M. alfredi* use of RS (Armstrong *et al*., 2020; Axworthy *et al*., 2019; Dewar *et al*., 2008; Germanov *et al*., 2019; Harris *et al*., 2021; Stewart *et al*., 2018). Further telemetry studies and direct assessments of zooplankton abundance and composition may be needed to identify the drivers of *M. alfredi* visitation to Závora and improve our understanding of them.

Mozambique and Australia are among few places in the world where *M. alfredi* live along an extended continental coastline, which may explain the observed transience of individuals at these locations (Armstrong *et al*., 2020; Venables, 2020). Long‐term monitoring of both of these *M. alfredi* populations show habitat use of an entire coastline where movement patterns may result from temporal shifts in productivity, as opposed to island habitats, which may have more reliable food sources in the area (Armstrong *et al*., 2020; Peel *et al*., 2019, Peel *et al*., 2020; Rohner *et al*., 2013; Venables, 2020). The longest point‐to‐point migration reported for an individual *M. alfredi* was 1150 km in Australia (Armstrong *et al*., 2019), whereas telemetry studies in Mozambique found rapid movements of up to 90 km in a single day (Venables *et al*., 2020). The Inhambane coastline consists of a narrow continental shelf with mesoscale, eddy‐driven upwelling in the Mozambican channel, which contributes to productivity, thus its fluctuation may drive *M. alfredi* movements up and down the coast (Quartly & Srokosz, 2004; Rohner *et al*., 2013). The support of Markovian emigration in the PRD models further implies that some *M. alfredi* individuals leave for multiple seasons and eventually return. Variations in movement and visitation patterns between the years could be attributed to oceanic processes that affect zooplankton patchiness and distribution including El Niño Southern Oscillation (ENSO) and/or dipole effects (Beale *et al*., 2019; Folt & Burns, 1999; Whitney & Crow, 2007).

The annual abundance of *M. alfredi* identified at this single reef in Závora is high when compared to aggregations in Hawaii (Axworthy *et al*., 2019; Deakos *et al*., 2011), the Seychelles (Peel, 2019) and Japan (Kashiwagi, 2014), and lower when compared to the seasonal, site‐specific, aggregation at Lady Elliot Island, Australia (Couturier *et al*., 2014). Previously published abundance estimates (2003–2012) from the Tofo region of the Inhambane Province (Marshall *et al*., 2011; Venables, 2020) were larger than our estimates for Závora, but with fewer overall resightings. Nonetheless, after 2013, Venables (2020) found <100 *M. alfredi* individuals to be using the reefs around Tofo, whereas in the present study abundances at RS were consistently >100 from 2017 to 2020. Abundance estimates between 2016 and 2021 varied noticeably, with 2017 and 2020 having greater capture rates compared to other primary periods. Such variation each year may be attributed to productivity shifts or ontogenetic factors, although further study is required to confirm this.

Both sexes displayed similar use of RS in contrast to many monitored locations where females are more frequently resighted (Marshall *et al*., 2011; Setyawan *et al*., 2018). The observed even sex ratio in this study supported preliminary findings by Venables (2020), but in contrast to the 61% female‐bias found in Tofo (Venables, 2020). Often when a greater geographic area is monitored with increased information on the metapopulation, even sex ratios have been reported, including in the Maldives and French Polynesia, or more uncommonly at a single site (Carpentier *et al*., 2019; Perryman *et al*., 2019; Stevens, 2016; Venables, 2020). Male *M. alfredi* were primarily mature at RS, with several juveniles that later returned as mature over the course of the study. The main difference in site use by the sexes was that specific mature females (*n* = 13) were resighted on 10 or more occasions, with some of these individuals encountered at RS over a duration of almost 11 years. Our findings of an aggregation of *M. alfredi* returning to this exact reef may reflect the importance of this site for sociality and/or courtship ritual (Perryman *et al*., 2021; Stevens *et al*., 2018; Thorburn *et al*., 2019).

An estimated average residence time of 4 days from the LIR analysis suggests *M. alfredi* individuals to visit the study site for a short period of time in peak season and then leave. The large ranges in standard error and 95% c.i. in the LIR analysis are likely due to the individual variability in sightings from the empirical data, and the variation in sightings year to year, which was also apparent in the PRD analysis. The residence time to RS was lower than *M. alfredi* populations around islands in French Polynesia (range 66–130 days) and Coral Bay, Australia (56 days); nonetheless, residence time out was lower than French Polynesia (range 59–117) and Coral Bay, Australia (92 days), suggesting that in Závora, individuals are more likely to move in and out of the study area even during peak season (Armstrong *et al*., 2020; Carpentier *et al*., 2019).

Challenging weather conditions, the logistics of operating in a remote location and resource availability contributed to uneven sampling effort throughout the study period. The authors accounted for this in the PRD analysis by modelling capture probability with an effect of sampling effort. Further limitation in sampling for the PRD included times when an individual was present at the aggregation but not photographed. Such limitations are characteristic of *M. alfredi* photo‐ID studies, including potential violations of model assumptions (*i.e*., survival probability being the same for all individuals) (Deakos *et al*., 2011; Couturier *et al*., 2014; Venables, 2020). Given the level of anthropogenic impact (Venables, 2020) and predation pressure (Marshall & Bennett, 2010b) affecting southern Mozambique may result in similar survivorship of this specific aggregation. Considering their longevity, this 6 year analysis is brief relative to the life span of *M. alfredi*. Nevertheless, the PRD in this context provided baseline estimations of the number of *M. alfredi* that use RS in Závora, Mozambique, an area which is currently unprotected.

More than 20 years of research along the Inhambane Province has identified the largest known *M. alfredi* population in Africa, yet with drastic declines in sightings, of up to 88% (Marshall *et al*., 2011; Rohner *et al*., 2013; Venables, 2020). This population is now stated to be of immediate conservation concern by local and international scientists (Peel, 2019; Rohner *et al*., 2013; Tibiriçá *et al*., 2011; Venables, 2020). *Mobula alfredi* is listed in Appendix II (2013) of the Convention for International Trade in Endangered Species (CITES) and in Appendices I and II (2014) of the Conservation of Migratory Species of Wild Animals (CMS). Nationally, manta species were protected under Mozambican law in 2017 (Law 5/2017) which banned fishing of CITES‐listed species; nonetheless, little was enforced (Boletim da Republica May 2017; Venables, 2020). As a vulnerable (Marshall *et al*., 2018) and economically important species (Venables *et al*., 2016), *M. alfredi* officially received national protection in 2021 (Boletim da República, 2020); nonetheless, along the Inhambane coastline, they remain under threat from indiscriminate netting and longlining, particularly in the south of the province (Marshall *et al*., 2011; Temple *et al*., 2018). To increase protection of this mobile species in Mozambique, it is essential to focus on priority habitats where they might be at risk, such as RS, where a seasonal inshore aggregation occurs every year. At present, the majority of protected critical habitat is concentrated in the north of the province in the Bazaruto Archipelago (Pelegrín *et al*., 2015). Although part of a single breeding population, photo‐ID and acoustic telemetry have indicated preferential habitat use to different sites, meaning that *M. alfredi* individuals using the northern regions do not show equal visitation to the southern regions of Tofo and Závora (Venables *et al*., 2020). Anthropogenic pressures from fishing continue to impact the southern *M. alfredi* in most of their home range, including Závora, which is at the southern extent of the area where they are most commonly encountered in Mozambique. Therefore, the authors recommend immediate, site‐specific protection of key habitats in the south, such as RS, as an essential step for conservation management. They also advocate for the design and implementation of a standalone marine‐protected area in Závora Bay to protect the larger critical habitat for elasmobranchs in this southern region of the Inhambane Province (O'Connor & Cullain, 2021). The Inhambane coast was declared a Mission Blue Hope Spot in 2022 in recognition of its diversity of threatened species, and the government of Mozambique has proposed to implement a large seascape‐type environmental protection area (EPA) from the Bazaruto Archipelago southwards towards Závora (Administração Nacional das Áreas de Conservação and Conservation International, 2020). Given the trajectory of the decline of the *M. alfredi* population along this coastline and the seasonal importance of this habitat, the authors advise the protection of Závora Bay be prioritised during this process.

## AUTHOR CONTRIBUTIONS

M.C. conceived the central idea of the article. Data collected by N.R.C. and Y.T. with some contributions by all authors. Analysis by M.C. with input by S.K.V. Written by M.C. with input from all other authors.

## Supporting information


**TABLE S1** List of individual *Mobula alfredi* (*n* = 21) never identified or resighted at Red Sands but identified at other dive sites off the coast of Závora, from 2010 to 2021

**TABLE S2** Parameter estimates, standard errors (s.e.) and 95% confidence intervals (c.i.) from the best‐fit model: capture probabilities (*p*) with sampling effort effect between each primary period; Markovian emigration (γ″ γ′) between each primary period, and rate of constant apparent survival (φ) across all periods

**TABLE S3** Parameter estimates from the best supported models in the lagged identification rates (LIR) analysis at Red Sands, Zavora, Mozambique, 2016–2021

**FIGURE S1** Manta Za288 pregnant in 2017 (top left) and not visibly pregnant in 2018 (top right). Manta Za160 not visibly pregnant in 2017 (bottom left) and pregnant in 2018 (bottom middle) and 2021 (bottom right). Photography credit: MAR Expeditions (top left), Nakia Cullain (top right), Anna Flam (bottom left), Nakia Cullain (bottom middle) and Nakia Cullain (bottom right)
