# Supplementary material for: Evidence of Závora Bay as a critical site for reef manta rays, Mobula alfredi, in southern Mozambique
Source: J Fish Biol. 2022 Jul 23;101(3):628–39. doi: 10.1111/jfb.15132 (PMC9544570; doi:10.1111/jfb.15132)
Supplement: Supplementary file 1 — TABLE S1 List of individual Mobula alfredi (n = 21) never identified or resighted at Red Sands but identified at other dive sites off the coast of Závora, from 2010 to 2021 [file JFB-101-628-s001.pdf]

Table S1. List of individual rays (n=21) never identified or resighted at Red Sands but identified at other dive sites off the coast of Závora, from 2010-2021.

ID	Sex	Site identified	No. of sightings
Za003	U	Area 51	1
Za007	U	Area 51	1
Za009	M	Area 51	1
Za011	U	Area 51	1
Za012	F	Area 51	1
Za020	F	Klipfontein	1
Za021	M	Area 51	1
Za023	F	Area 51	1
Za024	M	Area 51	1
Za041	M	Great Wall South	1
Za109	F	Pelagic/Závora	1
Za139	U	Area 51	1
Za156	M	Pelagic/Závora	1
Za206	F	Dean's	1
Za240	F	Yogi's	1
Za299	M	Pelagic/Závora	1
Za490	F	Area 51	1
Za514	F	Pelagic/Závora	1
Za588	M	Yogi's	1
Za641	M	Deep Reef South	1
Za643	F	Dean’s	1
