# Supplementary material for: Evidence of Závora Bay as a critical site for reef manta rays, Mobula alfredi, in southern Mozambique
Source: J Fish Biol. 2022 Jul 23;101(3):628–39. doi: 10.1111/jfb.15132 (PMC9544570; doi:10.1111/jfb.15132)
Supplement: Supplementary file 2 — TABLE S2 Parameter estimates, standard errors (s.e.) and 95% confidence intervals (c.i.) from the best‐fit model: capture probabilities (p) with sampling effort effect between each primary period; Markovian emigration (γ″ γ′) between each primary period, and rate of constant apparent survival (φ) across all periods [file JFB-101-628-s003.pdf]

Table S2. Parameter estimates, standard errors (SE), and 95% confidence intervals (CI) from the best-fit model: capture probabilities (p) with sampling effort effect between each primary period; Markovian emigration (γ'' γ') between each primary period, and rate of constant apparent survival (φ) across all periods.

Period	*p*	SE	95% CI	γ''	SE	95% CI	Φ^{Sex}	SE	95% CI
2016-17	0.158	0.011	0.137-0.181	0.506	0.123	0.281-0.729	-	-	-
2017-18	0.475	0.030	0.417-0.535	0.714	0.047	0.615-0.796	-	-	-
2018-19	0.293	0.016	0.262-0.326	0.617	0.075	0.464-0.749	-	-	-
2019-20	0.182	0.012	0.160-0.206	0.324	0.090	0.177-0.518	0.848^{Male}	0.087	0.597-0.954
2020-21	0.688	0.041	0.602-0.762	0.852	0.035	0.771-0.909	0.823^{Female}	0.083	0.602-0.935
