# Supplementary material for: Evidence of Závora Bay as a critical site for reef manta rays, Mobula alfredi, in southern Mozambique
Source: J Fish Biol. 2022 Jul 23;101(3):628–39. doi: 10.1111/jfb.15132 (PMC9544570; doi:10.1111/jfb.15132)
Supplement: Supplementary file 3 — TABLE S3 Parameter estimates from the best supported models in the lagged identification rates (LIR) analysis at Red Sands, Zavora, Mozambique, 2016–2021 [file JFB-101-628-s002.pdf]

Table S3. Parameter estimates from the best supported models in the LIR analysis at Red Sands, Zavora, Mozambique, 2016-2021.

Model	Model description	ΔQAIC	N	Residence time in	Residence time out	Mortality/ Permanent emigration
F	Emigration + reimmigration (*a1=N*; a2=Res time in; a3=Res time out	0.95	103.44	68.56	90.66	-
H	Emigration + reimmigration + mortality a1=N; a2= Res time in; a3=Res time out; a4=Mortality	0.00	57.62	4.41	9.95	0.0003
