# Supplementary material for: Evidence of Závora Bay as a critical site for reef manta rays, Mobula alfredi, in southern Mozambique
Source: J Fish Biol. 2022 Jul 23;101(3):628–39. doi: 10.1111/jfb.15132 (PMC9544570; doi:10.1111/jfb.15132)
Supplement: Supplementary file 4 — FIGURE S1 Manta Za288 pregnant in 2017 (top left) and not visibly pregnant in 2018 (top right). Manta Za160 not visibly pregnant in 2017 (bottom left) and pregnant in 2018 (bottom middle) and 2021 (bottom right). Photography credit: MAR Expeditions (top left), Nakia Cullain (top right), Anna Flam (bottom left), Nakia Cullain (bottom middle) and Nakia Cullain (bottom right) [file JFB-101-628-s004.pdf]

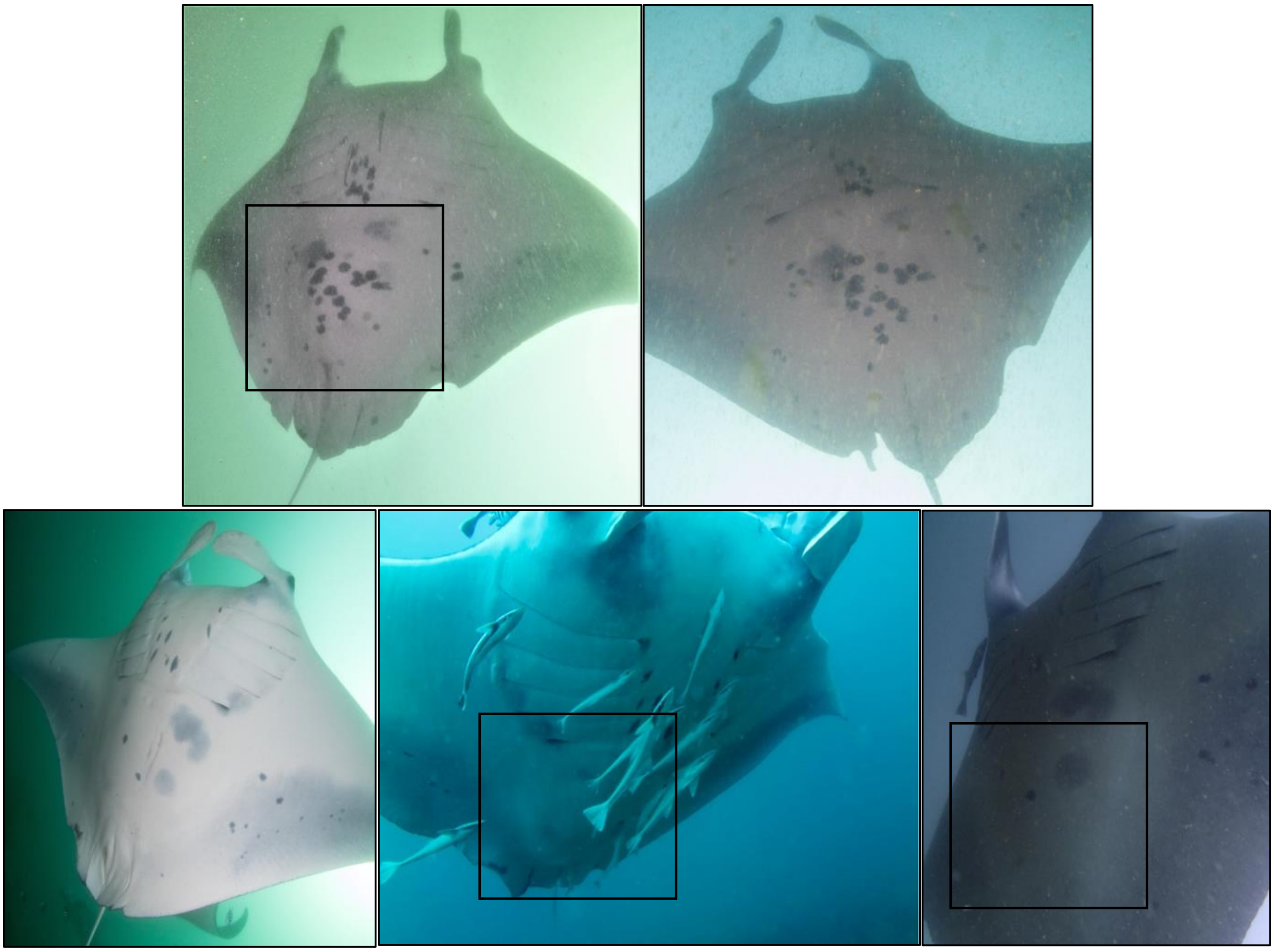

Figure S1. Manta Za288 pregnant in 2017 (top left) and not visibly pregnant in 2018 (top right). Manta Za160 not visibly pregnant in 2017 (bottom left), pregnant in 2018 (bottom middle) and 2021 (bottom right) Photography credit: MAR Expeditions (top left), Nakia Cullain (top right), Anna Flam (bottom left), Nakia Cullain (bottom middle), Nakia Cullain (bottom right).
